# CXCR4 uses STAT3-mediated slug expression to maintain radioresistance of non-small cell lung cancer cells: emerges as a potential prognostic biomarker for lung cancer

**DOI:** 10.1038/s41419-020-03280-5

**Published:** 2021-01-07

**Authors:** Jeong-Yub Kim, Hee-Jin Kim, Chan-Woong Jung, Tae Sup Lee, Eun Ho Kim, Myung-Jin Park

**Affiliations:** 1 Radiation Therapeutics Development Team, Division of Radiation Cancer Science, Korea Institute of Radiological and Medical Sciences, Seoul, Korea; 2 School of Biomedical Science, Korea University, Seoul, Korea; 3 Department of Life Sciences, Korea University, Seoul, Korea; 4 Division of RI Application, Korea Institute of Radiological and Medical Sciences, Seoul, Korea; 5 Department of Biochemistry, School of Medicine, Daegu Catholic University, 33, 17-gil, Duryugongwon-ro, Nam-gu, Daegu, 42472 Korea

**Keywords:** Radiotherapy, Prognostic markers

## Abstract

Lung cancer is one of the most common reasons for cancer-induced mortality across the globe, despite major advancements in the treatment strategies including radiotherapy and chemotherapy. Existing reports suggest that CXCR4 is frequently expressed by malignant tumor and is imperative for vascularization, tumor growth, cell migration, and metastasis pertaining to poor prognosis. In this study, we infer that CXCR4 confers resistance to ionizing radiation (IR) in nonsmall cell lung cancer (NSCLC) cells. Further, on the basis of colony forming ability, one finds that drug-resistant A549/GR cells with improved CXCR4 expression exhibited more resistance to IR than A549 cells evidenced along with a reduction in the formation of γ-H2AX foci after IR. Transfection of shRNA against CXCR4 or treatment of pharmacological inhibitor (AMD3100) both led to sensitization of A549/GR cells towards IR. Conversely, the overexpression of CXCR4 in A549 and H460 cell lines was found to improve clonogenic survival, and reduce the formation of γ-H2AX foci after IR. CXCR4 expression was further correlated with STAT3 activation, and suppression of STAT3 activity with siSTAT3 or a specific inhibitor (WP1066) significantly stymied the colony-forming ability and increased γ-H2AX foci formation in A549/GR cells, indicating that CXCR4-mediated STAT3 signaling plays an important role for IR resistance in NSCLC cells. Finally, CXCR4/STAT3 signaling was mediated with the upregulation of Slug and downregulation of the same with siRNA, which heightened IR sensitivity in NSCLC cells. Our data collectively suggests that CXCR4/STAT3/Slug axis is paramount for IR resistance of NSCLC cells, and can be regarded as a therapeutic target to enhance the IR sensitivity of this devastating cancer.

## Introduction

With a high death burden across the globe, lung cancer has emerged as a major healthcare problem. Small cell lung cancer (SCLC) and nonsmall cell lung cancer (NSCLC) account for up to 87% of lung cancer cases, thus constituting most frequent types of cancers^1^. More specifically, a 15% survival rate is seen in NSCLC patients. Despite employing numerous interventions like chemotherapy and radiotherapy, no such significant improvement is marked in the survival rate of the patients. This indicates a vast knowledge gap on the response of condition to various interventions and treatments as along with its tumorigenesis^2^. While chemotherapy remains the preferred option for treatment of NSCLCs with the exception of surgery; radiotherapy is the secondary option that continues to be one of the main treatment modality for those with independent NSCLC or with another form of treatment such as chemotherapy^3^. Notwithstanding the progress in radiation techniques, the survival rate of patients still needs improvement. A considerable number of patients are known to witness either local-regional recurrence or new cases of primary cancer after radiotherapy. For this reason it is necessary to develop radiotherapeutic strategies by focusing the crucial mechanism(s) for lung cancer’s radioresistance so as to improve the treatment outcomes.

The existence of cancer stem cells (CSCs) that trigger tumor heterogeneity are considered to be one of the most common reasons behind therapeutic failure after drastic radiotherapy and chemotherapy. Initially, lung-cancer-related CSCs (LCSCs) were found in the subpopulation of cells with CD133 on their cell surface extracted from the tissues of patients, both in SCLC and NSCLC cases^4^. LCSCs expressed exaggerated levels of embryonic stem cell factor, Oct4, and Sox2, followed by drug pumping protein, ABCG2, all these factors are deemed to be responsible for self-renewal and chemoresistance, respectively^5,6^. Furthermore, aldehyde dehydrogenase-positive lung cancer cells and urokinase plasminogen activator receptor-positive also exhibit features of CSCs^7^. According to various reports, lung cancer cells that are resistant to ionizing radiation (IR) and drugs which exhibit CSCs characteristics are able to express several epithelial–mesenchymal transition makers and CSCs^8^. It is for this reason that it is necessary to target LCSCs in order to augment the clinical outcomes of lung cancer patients.

CXCR4 (chemokine (C-X-C motif) receptor 4) is a receptor for a chemokine stromal-derived growth factor-1α (SDF-1α), also known as CXCL12. Existing reports suggest that malignant tumors widely express CXCR4, which a paramount factor responsible for rapid growth, metastasis, and vascularization, along with poor prognosis. CXCR4/SDF-1α axis seemingly pertains to the NSCLC’s metastatic potential^9^. Activation of CXCR4 signaling increases the migration and invasion of NSCLC cells and blockade of this signaling reverses the effect in vitro and suppresses metastatic activity of these cells in vivo using neutralizing antibody^10^. CXCR4 is also called CSC marker since CSCs have the high level of CXCR4 expression on their surface^11,12^. Our previous study also found that CXCR4 is a functional LCSC marker for maintenance of stemness and tumorigenesis in NSCLC cells^13^. By employing transfection with antisense nucleotide for CXCR4 or blockade of CXCR4/SDF-1α axis with pharmacological inhibitor or neutralizing antibody, we concluded CXCR4 as a crucial factor for the maintenance of stemness, tumorigenesis and IR resistance in NSCLC cells^13^.

In the current study, we aim to discern the molecular mechanism of CXCR4-mediated IR resistance in NSCLC cells. Our results showed that CXCR4 signaling was proven to be crucial for IR-induced DNA damage repair in NSCLC cells. CXCR4 signaled to STAT3/Slug axis thereby reduced DNA damage caused by IR and increased clonogenic survival in NSCLC cells. Therefore, our results offer compelling evidence that targeting CXCR4 and STAT3/Slug signaling would be useful for enhancing IR sensitivity in NSCLC.

## Materials and methods

### Antibodies and reagents

Antibody against H2AX and γ-H2AX were obtained from MilliporeSigma (Burlington, MA). CXCR4 was purchased from LS Bioscience (Seattle, WA). Antibody such as p-STAT3 (S727) and β-actin were purchased from Santa Cruz Biotechnology (Santa Cruz, CA). p-STAT3 (Y705) and Slug were obtained from Cell Signaling Biotechnology (Denvers, MA). AMD3100 and WP1066 were obtained from Sigma-Aldrich (St. Louis, MO). SDF-1α was purchased from R&D systems (Minneapolis, MN).

### Cell culture

NSCLC cell lines (A549 and H460) were obtained from ATCC (Manassas, VA). A549/GR cells were established as described previous reports^14^. All cell lines were cultured in RPMI1640 (Welgene, Gyeongsan-si, Gyeongsangbuk-do, Korea) supplemented with 2 mM glutamine, 1 mM sodium pyruvate, 100 U/ml penicillin, 100 μg/ml streptomycin (Welgene) and 10% fetal bovine serum (FBS; GIBCO-Thermo Fisher Scientific, Waltham, MA) in a humidified incubator containing 5% CO_2_ at 37 °C.

### Knockdown or overexpression of CXCR4

For knockdown of CXCR, pGFP-C-shLenti and pGFP-C-shLenti CXCR4 vector were obtained from Origene (Rockville, MD). For overexpression of CXCR4, pQCXIP retrovirus vector was obtained from Takara Bio USA (Mountain View, CA) and CXCR4 was subcloned into the vector. Production of viruses was done by transfection of 293T cells with viral vectors and packaging mix using TransIT-X2 (Mirus Bio LLC, Madison, WI). After 48 h of transfection, viral supernatants were collected, filtered, and stored at deep freezer with polybrene (8 μg/ml; Sigma-Aldrich) until use.

### Transfection of small interfering RNA (siRNA)

Cells were transfected with a pool of siRNAs against CXCR4: 5′-AUCACGUAAAGCUAGAAA-3′ and 5′-GGGAUCAUUUCUAGCUUU-3′; STAT3: 5′-UCCAGUUUCUUAAUUUGUUGACGGGUU-3′ and 5′- GGCCAUGAACUUGACAAUAUCUGCUUU-3′, Slug: 5′-GAGAGAUUAUCUAUGCAUAAACAGCUU-3′ and 5′-ACCAGCAUUUCUAUACCACUUUGGGUU-3′ (Integrated DNA Technologies, Coralville, IA) at a final concentration of 20 nM using G-fectin (Genolution, Seoul, Korea).

### Semiquantitative reverse transcriptase-polymerase chain reaction (sqRT-PCR) and quantitative RT-PCR (qRT-PCR)

High-quality total RNA was isolated from cells by using TRIsure (BIOLINE, London, UK) as described in the manufacturer’s protocol. Complimentary DNA was synthesized using the cDNA synthesis kit (BIOLINE). Oligonucleotide primer sequences for sqRT-PCR used were as follows: CXCR4 forward: 5′-AATCTTCCTGCCCACCATCT-3′, CXCR4 reverse: 5′-GACGCCAACATAGACCACCT-3′; STAT3 forward: 5′-TAATGAAAAGTGCCTTTGTGG-3′, STAT3 reverse: 5′-TGACCAGCAACCTGACTTTAG-3′, Slug forward: 5′-TGTGACAAGGAATATGTGAGCC-3′, Slug reverse: 5’-TGAGCCCTCAGATTTGACCTG-3′, GAPDH forward: 5′-TGGTGAAGGTCGGTGTGAAC-3′, GAPDH reverse: 5′-TTCCCATTCTCAGCCTTGAC-3′.

cDNAs were analyzed by qPCR (CFX96 Touch^TM^ Realtime detection system, Biorad, Hercules, CA) using SYBR (BIOLINE). Samples were assayed in triplicate for each gene and relative expression was calculated by the ∆∆Ct method (Applied Biosystems, Foster city, CA). Oligonucleotide primer sequences for qRT-PCR used were as follows: CXCR4 forward: 5′-TCAACCTCTACAGCAGCGTTCTCTT-3′, CXCR4 reverse: 5′-TGTTGGTGGCGTGGACAAT-3′. The qRT-PCR primers of STAT3 and Slug were the same as sqRT-PCR primers.

### Western blot analysis

Cells were lysed in RIPA buffer (50 mM Tris-HCl (pH 7.4), 100 mM NaCl, 5 mM EDTA, 0.5% Nonidet P-40, phosphatase inhibitor cocktail setII (MilliporeSigma), and a protease inhibitor cocktail tablet (Roche, Basel, Switzerland). The protein contents were determined using the protein assay reagent (Bio-Rad, Hercules, CA). The proteins were separated using 8–12% SDS-PAGE gels and transferred to a nitrocellulose membrane. The membranes were blocked with 5% skim milk in TBST (20 mmol/l Tris‑HCl (pH 7.6), 137 mmol/lNaCl and 0.01% Tween-20) for 1 h at room temperature (RT) and then incubated with the indicated primary antibodies overnight at 4 °C with gentle shaking. After extensive washing with TBST, the membrane was developed with a peroxidase-conjugated secondary antibody for 2 h at RT. After washing three times with TBST for 10 min, membranes were visualized by enhanced chemiluminescence (Amersham, Pittsburgh, PA) according to the manufacturer’s protocol.

### Irradiation and clonogenic assay

For measuring IR sensitivity of NSCLC cells, cells were seeded in 6-well plates (1 × 10^3^ cells/ml) and exposed to γ-rays from a ^137^Cs γ-ray source (BIOBEAM8000, 2.6 Gy/min, Gamma-service Medical GmbH, Leipzig, Germany) at the indicated dose rate. After 7 days of incubation, spheres were attached by adding 10% FBS, washed with PBS, and stained with 0.05% crystal violet dissolved in 20% methanol. After washing three times with distilled water, colonies were counted.

### Immunocytochemistry (ICC) and γ-H2AX foci assay

Cells were seeded in a chamber slide with DMEM supplemented with 10% FBS. The next day, the cells were fixed with 4% paraformaldehyde for 10 min and washed with PBS three times. Then, the cells were incubated in blocking solution (5% BSA and 0.5% Triton X-100 in PBS) for 1 h at RT. The cells were stained with primary antibodies (γ-H2AX) in blocking solution (1:100) for 2 h and washed with PBS three times. Then, the cells were incubated with Alexa Fluor 594-labeled goat anti-mouse (Bethyl Laboratories, Montgomery, TX) secondary antibodies (1:1000) for 1 h. Nuclei were stained with DAPI and stained cells were viewed under a confocal laser-scanning microscope. γ-H2AX foci were determined in at least 50 cells.

### Immunohistochemistry (IHC)

IHC was performed to assess the expression of CXCR4, p-STAT3 (S705), and Slug in tumor xenograft sections and tissue array slide purchased from US Biomax (LC1921b.; Rockville, MD, USA). IHC was performed using antibodies against CXCR4, p-STAT3 (S705), and Slug (1:100), and secondary antibodies conjugated with the fluorescent dyes Alexa 488 or Alexa 555 (1:400; Jackson Labs). Tumor sections positively stained areas were evaluated with ImageJ software (http://imagej.net/).

### Cell cycle analysis

Cells were resuspended in 100% cold EtOH and chilled on ice overnight. The cells were then washed with PBS and stained with PBS containing 50 μg/ml PI, 10 μg/ml ribonuclease A, and 0.05% Triton X-100 for 40 min in the dark. After centrifugation, cells were resuspended in PBS. Flow cytometry analysis was performed on FACS Calibur (Becton Dickinson).

### Animal study

All of the experiments were conducted using protocols and conditions approved by the Institutional Animal Care and Use Committee in KIRAMS. Athymic BALB/c female mice (Nara Biotech, Seoul, Korea) were subcutaneously injected with A549/GR cells (5 × 10^6^ cells in PBS) in right thigh. A549/GR tumor bearing mice were randomized into four groups (*n* = 6–7). Treatment was performed with AMD3100 alone, irradiation alone, AMD3100 combined with irradiation, or control. Treatment with AMD3100 was started at −1 day before irradiation and maintained for 7 days. Control or AMD3100 treated group implanted with 7 days 0.5 μl/h osmotic pump (Alzet, Cupertino, CA) loaded with saline or 25 mg/ml of AMD3100 before one day irradiation. Irradiation was performed with X-ray unit operated at 260 kVp with a dose rate of 2 Gy/min (10 mA with added filtration of 2 mm copper, distance from X-ray source to the target of 41 cm). Tumor volume (mm^3^) was calculated using following formula: long diameter × (short diameter)^2^× 0.5. Tumor growth rate was compared by using Tumor volume doubling time (TDT). Body weight of the mice was monitored during all the treatments. No significant losses in body weight (less than 10%) were observed.

### Preparation of ^64^Cu-AMD3100

AMD3100 was purchased from Sigma-Aldrich, and ^64^Cu was produced at KIRAMS by 50 MeV cyclotron irradiation using methods reported^15^. [^64^Cu]AMD3100 was prepared according to literature method^16^. Briefly, 500 ng of AMD3100 were added to 37 MBq of ^64^CuCl_2_ buffered with 1 M sodium acetate buffer (pH 6.5) and incubated at 60 °C for 40 min. Quality control was performed by instant thin layer chromatography-silica gel (ITLC-sg, Pall, USA) with a mobile phase of 20 mM citrate buffer pH 5 with 50 mM EDTA.

### Receptor binding assay of [^64^Cu]AMD3100

A549 and A549/GR cells were seeded in 6-well plate (5 × 10^5^ cells/well) and incubated with 18.5 kBq/ml of [^64^Cu]AMD3100 for 1 h in incubation buffer (Hank’s balanced salt solution with 0.25% BSA). After incubation, cells were triple washed with incubation buffer and detached, cell bound radioactivity was determined in a gamma counter (Wizard 2480, Perkin-Elmer, USA). Radioactivity value was converted to percentage of added radioactivity dose (%AD) per million cells. Experiments were performed in triplicate.

### PET/CT imaging of [^64^Cu]AMD3100

Athymic BALB/c female mice (Nara Biotech) were subcutaneously injected with A549 or A549/GR cells (5 × 10^6^ cell in PBS) in right flank. After 24 days, PET/CT images were obtained by using Inveon PET/CT system (Simens Preclinical Solution, Germany). Before the injection of radiotracer, mice were anesthesized with 3% isofluorane and maintained under 2% isofluorane. Whole body imaging was acquired for 30 min static scan at 90 min post injection of [^64^Cu]AMD3100 (11.1 MBq). The percentage of injected dose per gram (%ID/g) value was calculated previously reported method^17^. Data were analyzed using Inveon Research Workplace software.

### Statistics analysis

Data are presented as mean ± SEM of at least three independent experiments. Differences were analyzed using the Student’s *t*-test and were considered significant at *P < 0.05, **P < 0.01, ***P < 0.005. Statistical analysis and graphing were performed using the Microsoft Excel 2013 and GraphPad Prism 6.0 software (GraphPad software).

## Results

### A549/GR cells are more resistant to IR than parent A549 cells

**Fig. 1:**
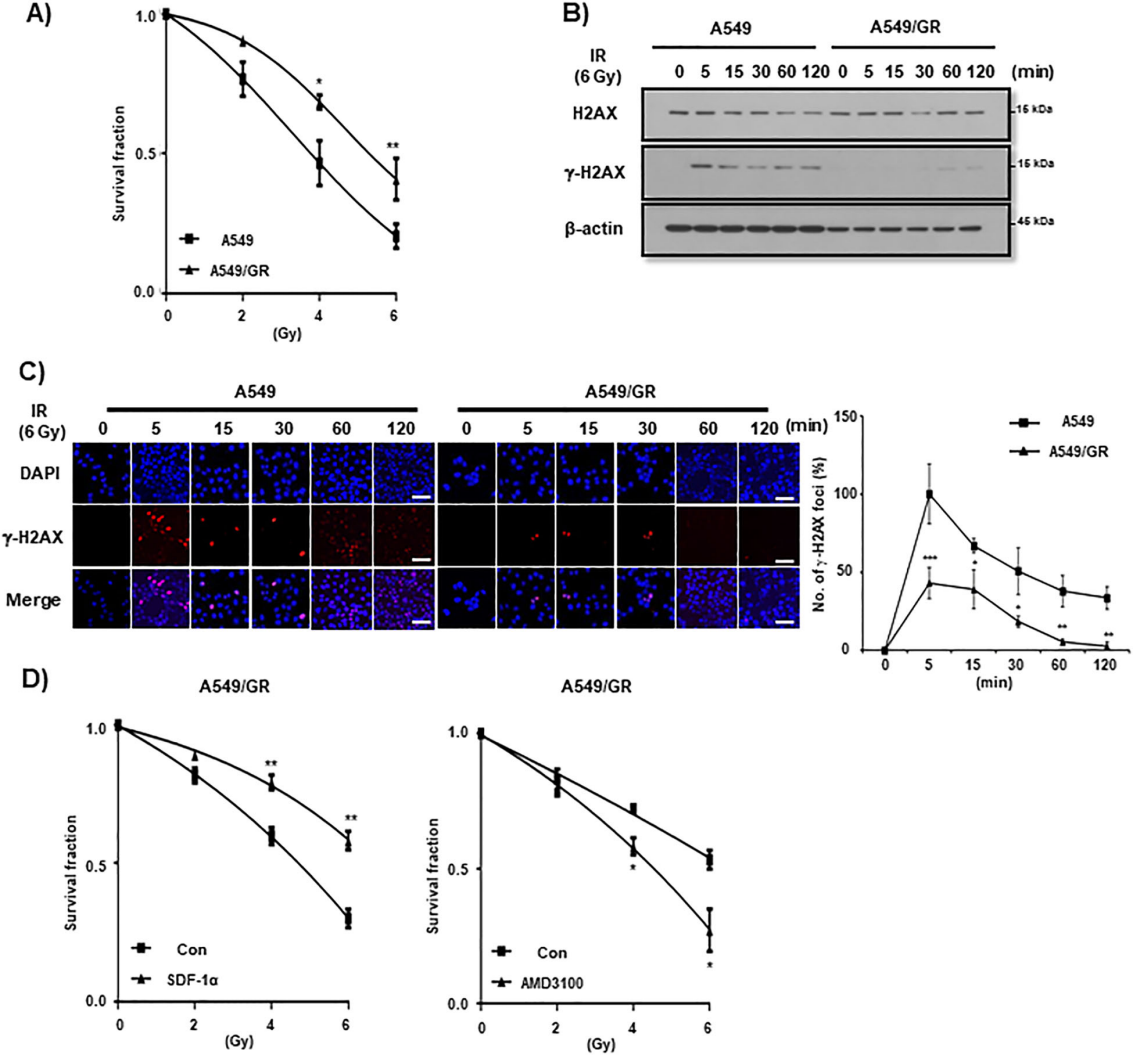
Drug resistant A549/GR cells are more resistant to IR than parent A549 cells. **A** IR clonogenic assay of A549 and A549/GR cells. Cells (2.5 × 10^2^) were exposed to IR at various doses described in the Figure. IR-exposed cells were cultured in complete medium and evaluated colony-forming activity at 10 days after IR exposure. **B** Western blot analysis of A549 and A549/GR cells. Cells were seeded in complete medium. Next day, cells were exposed to IR at 6 Gy and lysed in lysis buffer at various time points indicated in the Figure. **C** γ-H2AX foci assay of A549 and A549/GR cells. ICC was performed with A549 and A549/GR cells using γ-H2AX antibody. The images were obtained from A549 and A549/GR cells exposed to 6 Gy of IR at the indicated time points described in the Figure (left). Graphs represented statistical analysis of ICC data (right). Nuclei were counterstained with DAPI (blue). White bar: 20 μm. **D** IR clonogenic assay of A549/GR cells treated with or without SDF-1α (100 ng/ml) (left) and AMD3100 (10 μM) (right). *P < 0.05, **P < 0.01, ***P < 0.005.

In our previous report, it was shown that CXCR4+ cells are more resistant to IR as compared to CXCR4− cells in A549/GR cells^13^. To verify this phenomenon, we first examined whether A549/GR cells having high level of CXCR4 expression on their surface are more resistant to IR than A549 parent cells. In clonogenic assay, A549/GR cells exhibited high colony forming ability following IR exposure in contrast to A549 cells (Fig. 1A). IR may cause DNA damage along with checkpoint kinases activation, which could be detected by employing assays for examining the role in recruiting and phosphorylation of histone H2AX at ser-139 (γ-H2AX)^18^. As shown in Fig. 1B, Western blot analysis showed that expression of γ-H2AX was lower in A549/GR cells than A549 cells after IR. ICC data revealed that IR-induced formation of γ-H2AX foci started at 5 min and persisted upto 2 h in A549 cells. IR also induced a considerable increase of γ-H2AX foci in A549/GR cells at five min, however, it was much lower than A549 cells and started to be resolved at 15 min time point and almost completely disappeared at an h after IR (Fig. 1C). In the cell cycle analysis, IR exposure led to a higher increase in the subG1 fractions. This indicates more cell deaths in A549 cells than in A549/GR cells (Supplemental Fig. 1A). Accordingly, Western blot analysis revealed that IR exposure caused a higher expression of cleaved caspase 3 (cCas3) and cleaved Parp (cParp) in A549 cells than in A549/GR cells (Supplemental Fig. 1b). These data suggest that A549/GR cells have higher activity of repairing DNA damage by IR evidenced by γ-H2AX foci assay than A549 parent cells, which might lead to enhanced clonogenic survival of the latter after IR. To investigate the functional role of CXCR4 signaling in the survival activity of NSCLC cells from IR exposure, we treated the cells (A549, A549/GR, and H460) with SDF-1α, a ligand of CXCR4 and AMD3100, a specific antagonist of CXCR4 and performed clonogenic survival assays. As shown in Fig. 1D (left panel), pretreatment of SDF-1α was found to improve clonogenic survival of A549/GR cells. Conversely, blockade of CXCR4 signaling with AMD3100 significantly suppressed colony-forming ability after IR in A549/GR cells (Fig. 1D, right panel). Clonogenic survival rate was also increased by SDF-1α and decreased by AMD3100, respectively in A549 and H460 cells after IR, although it was not statistically significant (Supplemental Fig. 2A, B). These data collectively suggest that activation of CXCR4 signaling is important for NSCLC cells to survive from the IR-induced DNA damage.

### CXCR4 knockdown sensitizes A549/GR cells to IR

**Fig. 2:**
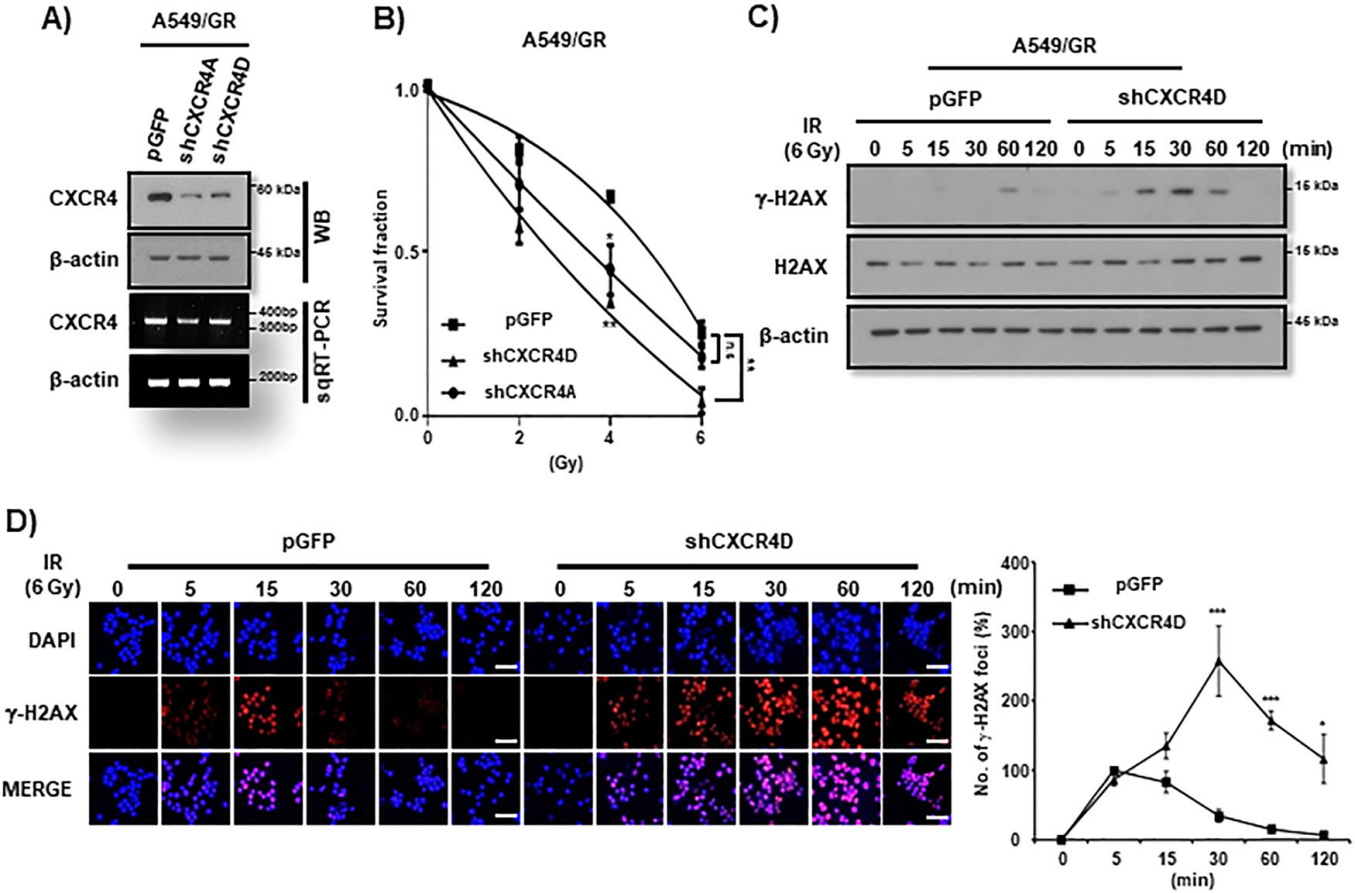
Knockdown of CXCR4 decreases the survival of A549/GR cells to IR. **A** Western blot (WB, upper) and RT-PCR (lower) analysis of CXCR4 in A549/GR cells transfected with shControl (pGFP) or shCXCR4A and D. **B** IR clonogenic assay of A549/GR cells transfected with shCXCR4D. Cells (2.5 × 10^2^) were exposed to IR at various doses described in the Figure. IR-exposed cells were evaluated colony-forming activity at 10 days after IR. *P < 0.05, **P < 0.01, ***P < 0.005. **C** Western blot analysis of A549/GR cells exposed to IR at 6 Gy at the indicated time points transfected with shControl (pGFP) or shCXCR4D. **D** γ-H2AX foci assay of A549/GR cells. ICC was performed with the cells transfected with shControl (pGFP) or shCXCR4D using γ-H2AX antibody after exposing to IR (6 Gy) at the indicated time points. Representative immunofluorescence images of A549/GR cells (left) and quantification of the assay (right). Nuclei were counterstained with DAPI (blue). White bar: 20 μm.

In order to ascertain the involvement of CXCR4 signaling in IR resistance of NSCLC cells, we performed the study dealing with the effects of loss of function of a CXCR4. To that end, we introduced shCXCR4 into A549/GR cells for knockdown the CXCR4 (Fig. 2A and Supplemental Fig. 3A). As shown Fig. 2B, knockdown of CXCR4 decreased clonogeinc survival activity of A549/GR cells to IR. Western blot analysis and γ-H2AX foci assay also revealed that targeting CXCR4 enhanced the expression of γ-H2AX (Fig. 2C), and caused the persistence of γ-H2AX foci formation upto 2 h investigation after IR in these cells (Fig. 2D), respectively. Additionally, cell cycle and Western blot analysis revealed that CXCR4 knockdown increased sub-G1 fractions and cCas3 and cParp expression A549/GR cells (Supplemental Fig. 3B, C).

### CXCR4 overexpression enhanced IR resistance in A549 and H460 NSCLC cells

**Fig. 3:**
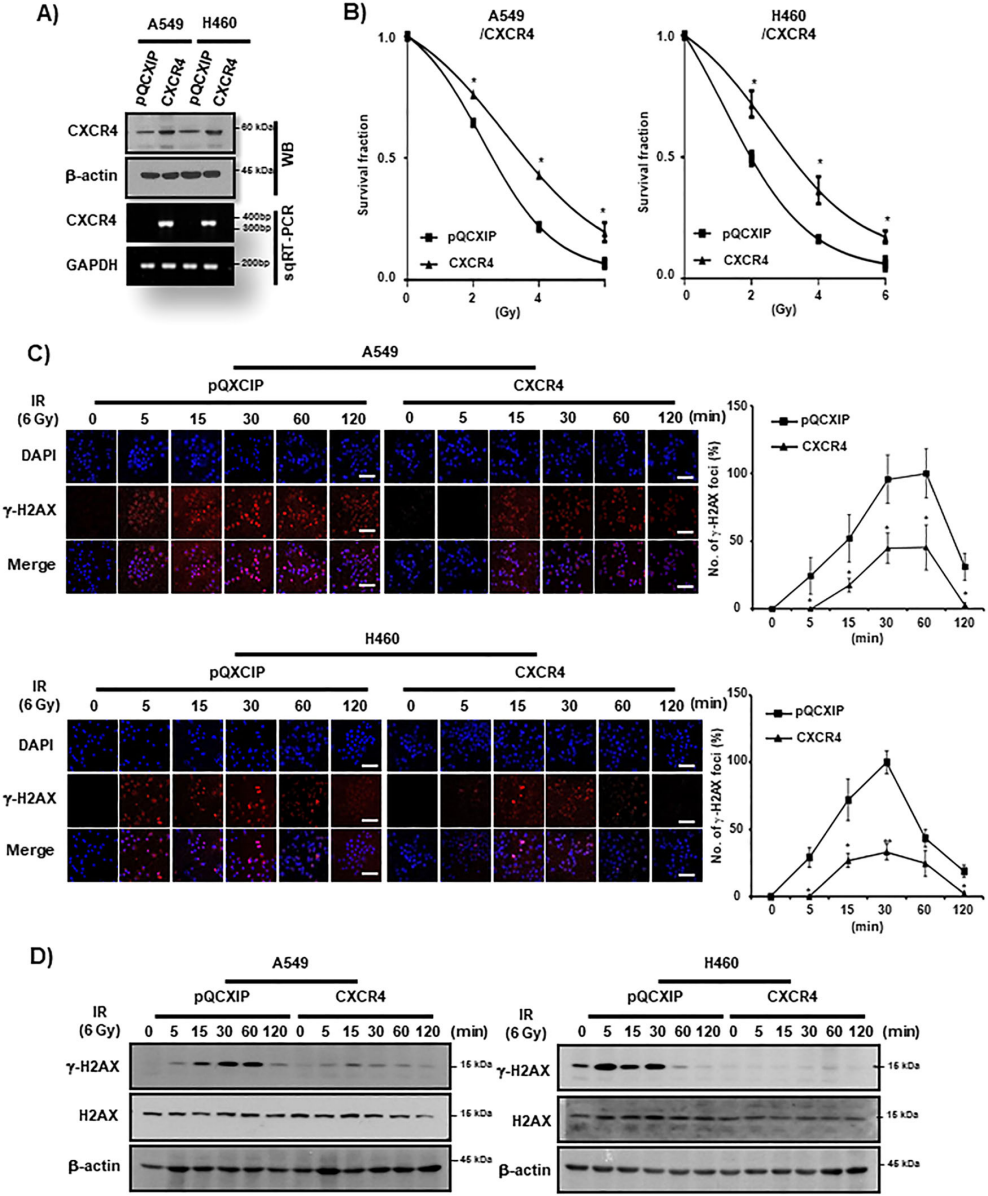
CXCR4 upregulation enhances IR resistance in NSCLC cells. **A** Western blot (WB, upper) and RT-PCR (lower) analysis of NSCLC cells (A549 and H460) transfected with CXCR4. β-actin and GAPDH were used as a loading control for Western blot and RT-PCR, respectively. **B** IR clonogenic survival assay of control vector (pQCXIP) and CXCR4 overexpressing (CXCR4) NSCLC cells. Cells were exposed to IR at the indicated dose in the Figure. **C** γ-H2AX foci assay of A549 (upper) and H460 (lower) cells. Cells were transfected either with control vector (pQCXIP) or pQCXIP-CXCR4 (CXCR4) and were exposed to IR (6 Gy) and fixed at the indicated time points in the Figure. Representative ICC images of γ-H2AX (left) and quantification of the results (right). Nuclei were counterstained with DAPI (blue). White bar: 20 μm. *P < 0.05, **P < 0.01. **D** Western blot analysis of A549 (left) and H460 (right) cells exposed to IR (6 Gy) at the indicated time points transfected with control vector (pQCXIP) or pQCXIP-CXCR4 (CXCR4). H2AX and β-actin were used as loading controls.

Next, we examined the IR resistance effect of CXCR4 by the study of gain of function of CXCR4. Overexpression of CXCR4 evidenced by Western blot analysis and RT-PCR (Fig. 3A and Supplemental Fig. 4A) in A549 and H460 cells significantly increased IR resistance of these cells in clonogenic assay (Fig. 3B). γ-H2AX foci assay also revealed enhanced foci resolution by CXCR4 expression in these cells (Fig. 3C). Western blot analysis clearly revealed that CXCR4 expression downregulated γ-H2AX expression by IR after overexpression of CXCR4 (Fig. 3D). Cell cycle and Western blot analysis also indicated that overexpression of CXC4 suppressed the increase of subG1 fractions and cCas3 and cParp expression in A549 and H460 cells (Supplemental Fig. 4B–D).

### CXCR4-mediated STAT3 activation controls the IR resistance in NSCLC cells

**Fig. 4:**
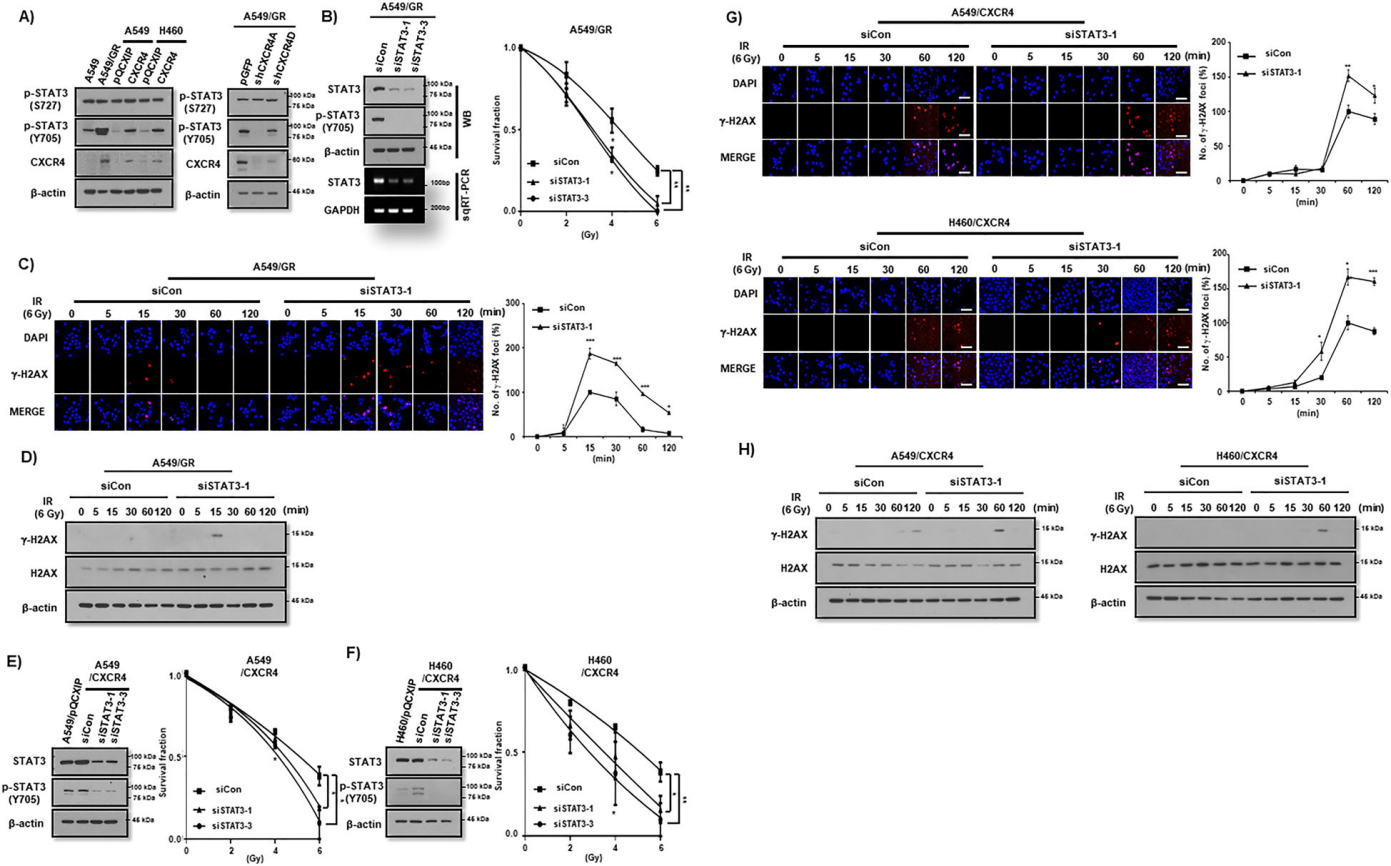
CXCR4-mediated STAT3 activation is critical for IR resistance of NSCLC cells. **A** Western blot analysis of phopho-STAT3 (Y705 and S727) in NSCLC cells (A549, H460, and A549/GR) transfected with the vectors described in the Figure. **B** Western blot (WB, upper) and RT-PCR (lower) analysis (left) and IR clonogenic survival assay (right) of A549/GR cells transfected with control siRNA (siCon) or siRNA against STAT3 (siSTAT3-1 and 3-2). **C**, **G** γ-H2AX foci assay of A549/GR (C) and A549/CXCR4 (upper) and H460/CXCR4 (lower) (**G**) cells. Cells were exposed to IR (6 Gy) and fixed at the indicated time points in the Figure. Representative ICC images of γ-H2AX (left) and quantification of the results (right). *P < 0.05, **P < 0.01, ***P < 0.005. White bar: 20 μm. **D**, **H** Western blot analysis of A549/GR (**D**) and A549/CXCR4 (left) and H460/CXCR4 (right) (**H**). Cells exposed to IR (6 Gy) at the indicated time points transfected with control siRNA (siCon) or siRNA against Stat3 (siSTAT3-1). **E**, **F** Western bot analysis of p-STAT3 (S727 and Y705) (left) and IR clonogenic survival assay (right) in A549 (**E**) and H460 (**F**) cells transfected with control siRNA (siCon) or siRNA against STAT3 (siSTAT3-1 and siSTAT3-3).

Previously, we found that CXCR4 signaled to STAT3 in order to maintain stemness in NSCLC cells^13^. Thus, we investigated the involvement of STAT3 in the CXCR4-mediated IR resistance of NSCLC cells. Overexpression of CXCR4 stimulated STAT3 phosphorylation, but knockdown of CXCR4 constitutively decreased phosphorylation of STAT3 at the tyrosine 705 (pY-STAT3), but not at the serine 727 sites (pS-STAT3) in NSCLC cells (Fig. 4A). siRNA-mediated knockdown of STAT3 (Fig. 4B, left panel and Supplemental Fig. 5A) significantly reduced clonogenic survival of A549/GR cell after IR (Fig. 4B, right panel). In the γ-H2AX foci assay, treatment of si-STAT3 led to increase the foci formation, and delay the resolution time after IR in A549/GR cells (Fig. 4C). Western blot analysis also showed the upregulation of γ-H2AX expression upon siSTAT3 transfection (Fig. 4D). Upon IR exposure, knockdown of STAT3 enhanced subG1 fractions and cCas3 and cParp expression in A549/GR cells (Supplemental Fig. 5B, C). In addition, transfection of siSTAT3 significantly reduced clonogenic survival of CXCR4 overexpressed NSCLC cells (Fig. 4E, F). γ-H2AX foci assay demonstrated that treatment of siSTAT3 resulted in reduced foci formation after IR in CXCR4 overexpressed A549 and H460 cells (Fig. 4G). Western blot analysis also showed elevated γ-H2AX expression after transfection of siSTAT3 in these cells (Fig. 4H). Finally, pretreatment of WP1066, a specific inhibitor of STAT3 decreased clonogenic survival of CXCR4-overexpressed NSCLC cells after IR (Supplemental Fig. 6). Taken together, IR stimulation of CXCR4 signals to STAT3 at the tyrosine 705 (pY-STAT3), which might be one of the major pathways for NSCLC cells to overcome IR-induced DNA damage.

### CXCR4/STAT3 signaling promotes IR resistance through Slug expression

**Fig. 5:**
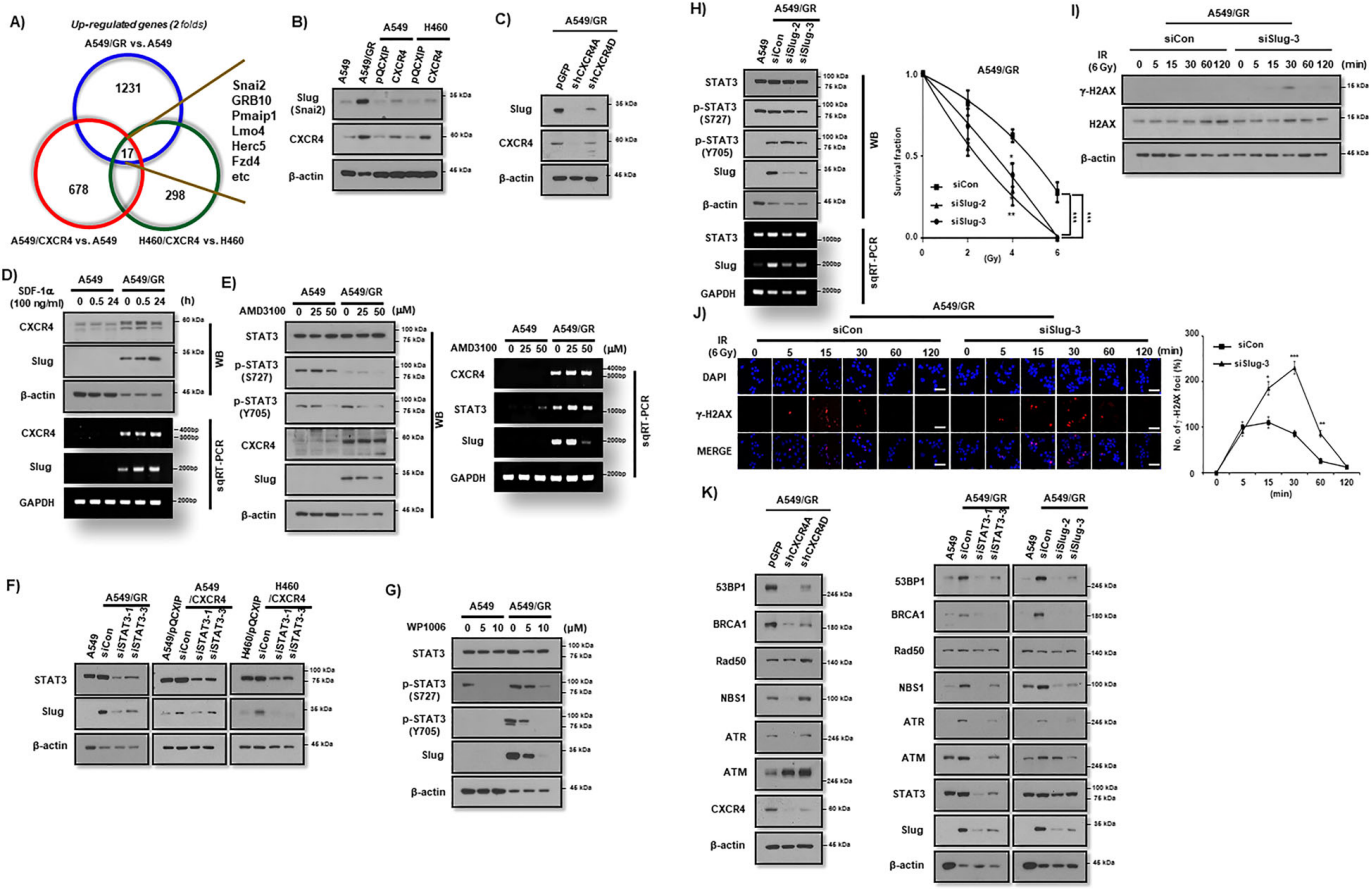
Upregulation of Slug is responsible for the IR resistance in CXCR4 overexpressed NSCLC cells. **A** Microarray of NSCLC cells for extracting commonly elevated genes between the groups described in the Figure. **B**–**G** Western blot (**B**–**H**) and RT-PCR (**D**, **E**, and **H**) analysis in NSCLC cells overexpressing CXCR4 (**B**), transfected with shCXCR4 (**C**), treated with SDF-1α (**D**), pretreated with AMD3100 (**E**), transfected with siSTAT3 (**F**), pretreated with WP1066 (**G**), and transfected with siSlug (**H**, left). Antibodies and RT-PCR primer used in the experiments are described in the Figure. β-actin and GAPDH were used as a loading control for Western blot and RT-PCR, respectively (**H**, right). IR clonogenic survival assay of A549/GR cells transfected with control siRNA (siCon) or siRNA against Slug (siSlug-2 and siSlug-3). **I** Western blot analysis of A549/GR cells transfected with siCon or siSlug-3. Cells were seeded in complete medium. Next day, cells were exposed to IR at 6 Gy, lysed in lysis buffer at various time points indicated in the Figure, and probed with antibodies indicated in the Figure. β-actin was used as a loading control. **J** γ-H2AX foci assay of A549/GR cells transfected with siCon or siSlug-3. ICC images were obtained from cells exposed to 6 Gy of IR at the indicated time points described in the Figure using γ-H2AX antibody (left). Graphs represented statistical analysis of ICC data (right). Nuclei were counterstained with DAPI (blue). *P < 0.05, **P < 0.01, ***P < 0.005. White bar: 20 μm. **K** Western blot analysis of A549/GR cells transduced with lentiviral pGFP or shCXCR4 (left) and transfected with siCon, siSTAT3, or siSlug (right). Antibodies used are described in the Figure.

Next, we performed microarray to find downstream effector molecule for CXCR4/STAT3 signaling that is responsible for IR resistance in NSCLC cells. With the criteria of *P* < 0.01 and |log2(FC) | ≥ 2, 1231 differentially expressed genes (DEGs) were identified when comparing A549/GR with A549, 678 DEGs were found when comparing A549/CXCR4 with control A549 and 298 DEGs when comparing H460/CXCR4 with H460. As presented in Fig. 5A, following construction of a Venn diagram, 17 DEGs were significantly differentially expressed among all three groups (Fig. 5A). Among these genes, we selected Snai2, also named Slug, as a possible effector molecule of CXCR4/STAT3 signaling, since several reports have shown the role of Slug in IR resistance of various cancer cells^13–19^. Western blot analysis revealed that Slug expression was upregulated in A549/GR cell line and CXCR4 overexpressing A549 and H460 cells in contrast to parent cells (Fig. 5B). Knockdown of CXCR4 suppressed Slug expression (Fig. 5C), whereas activation of CXCR4 signaling with SDF-1α increased Slug expression in A549/GR cells (Fig. 5D and Supplemental Fig. 7A). In addition, pretreatment of AMD3100 also inhibited the Slug expression in A549/GR cells (Fig. 5E and Supplemental Fig. 7B). Transfection of siSTAT3 suppressed Slug expression in A549/GR and CXCR4 overexpressing A549 and H460 cells lines (Fig. 5F). Similarly, STAT3 inhibitor (WP1006) inhibited the expression of Slug in A549/GR cells (Fig. 5G). Importantly, siRNA-mediated suppression of Slug significantly decreased clonogenic survival (Fig. 5H and Supplemental Fig. 7C) and increased γ-H2AX expression (Fig, 5I) and foci (Fig. 5J) in A549/GR cells after IR. Also, Knockdown of Slug increased IR-induced subG1 fractions and cCas3 and cParp expression in A549/GR cells (Supplementary Fig. 7D, E). Finally, transfection of siCXCR4, siSTAT3, and siSlug significantly suppressed the expression of DNA repair-related proteins, including 53BP1, BRCA1, NBS1, and ATR (Fig. 5K). Taken together, these data suggest that CXCR4/STAT3/Slug axis is crucial for IR resistance in NSCLC cells.

### Combination treatment of irradiation and AMD3100 reduced tumor growth rate

**Fig. 6:**
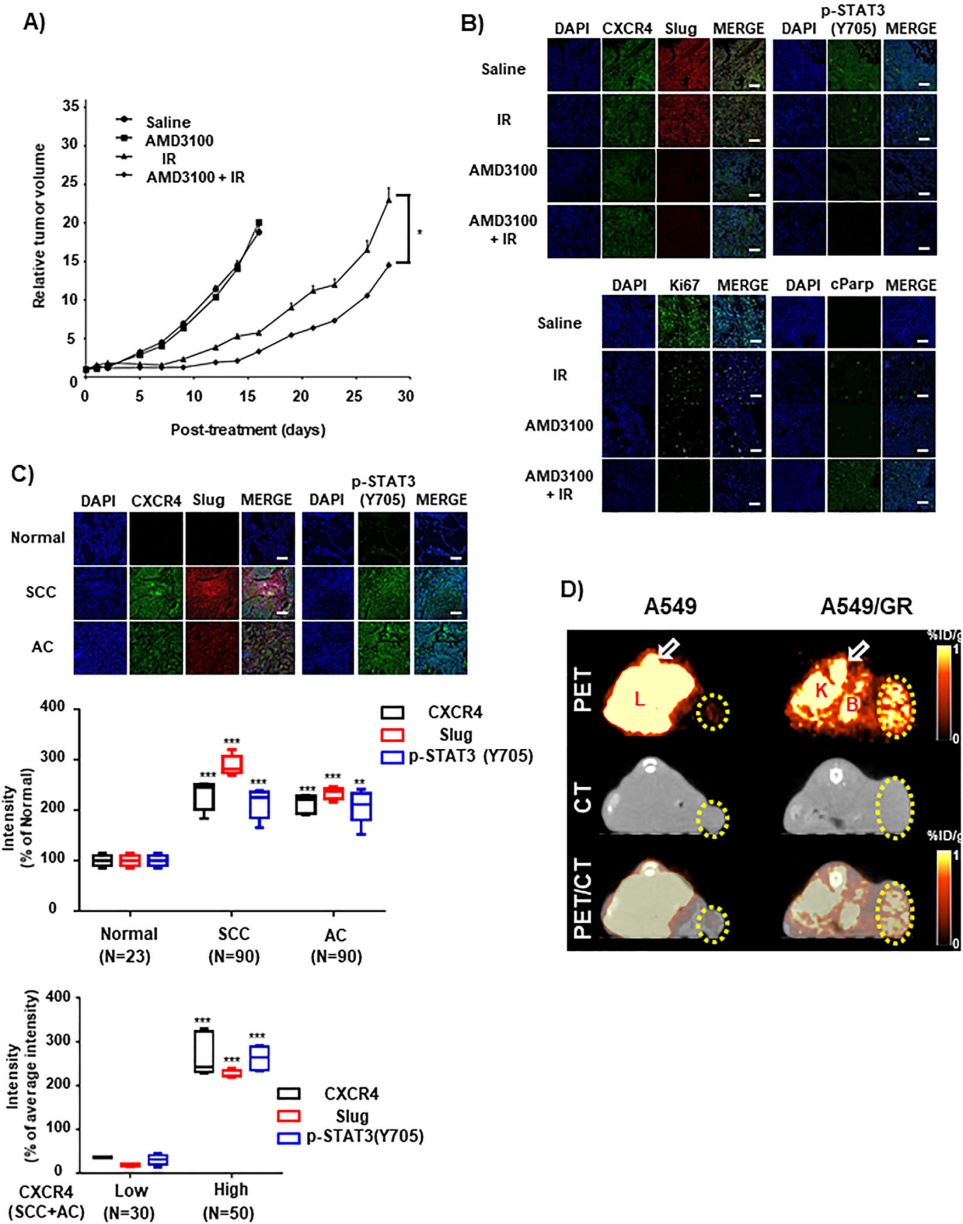
Treatment of CXCR4 inhibitor enhances IR sensitivity in vivo. **A** AMD3100 (5 mg/kg) was released from the alzet pump implanted beneath the skin on the top of the neck of mice. IR (10 Gy) was focally exposed to the tumor when it reached to volume of 100 mm^3^. Detailed experimental procedure was described in the “Materials and methods” section. **B** Representative illustration of IHC of xenograft tissues using antibodies shown in the Figure. **C** IHC of NSCLC patients tissue array using antibodies is indicated in the Figure. Representative picture of the staining (in the upper part) and quantification of the fluorescence intensity (middle and lower). Nuclei were stained with DAPI. SCC squamous cell carcinoma, AC adenocarcinoma. White bar 100 μm. **D** PET/CT imaging of CXCR4 expression in subcutaneous A549 or A549/GR tumor xenografts with [^64^Cu]AMD3100. Athymic mice bearing A549 or A549/GR xenografts on the right flank, were given ~11.1 MBq (300 μCi) of ^64^Cu-labeled radiotracers via tail vein injection, and PET/CT images were acquired. The representative transaxial PET, CT, and PET/CT sections of A549 or A549/GR tumors (yellow circle) from a [^64^Cu]AMD3100-injected mouse at 90 min postinjection. Specific accumulation of radioactivity in A549/GR over A549 is apparent. The bone marrow (arrow), liver, and kidney uptake of [^64^Cu]AMD3100 was also visualized. L liver, K kidney, B bladder. *P < 0.05.

Next, we evaluated the therapeutic effect of combination of IR (10 Gy) and AMD3100 in vivo. Tumor-bearing mice were infused with AMD3100 starting at −1 day before IR. AMD3100 has no significant effect on the growth of non-irradiated tumor (Fig. 6A). IR alone or AMD3100 and IR combination treatment reduced the tumor growth rate. However, AMD3100 and IR combination treatment further demonstrated a reduced tumor growth rate in contrast to irradiation alone (*P* < 0.05) (Fig. 6A). TDT in control and AMD3100 alone were similar, as 3.78 and 3.70 day, respectively. TDT in irradiation alone and combination treatment is 6.19 and 7.20 day, respectively. IR treatment showed approximately 1.6-times increased TDT, compared to control or AMD3100 alone. Combination (AMD3100 + IR) treatment showed 1.16-fold increased TDT, compared to IR alone. IHC of xenograft tissue demonstrated that AMD3100 and the combination of AMD3100 and IR significantly suppressed p-STAT3 and Slug in comparison to saline or IR alone sample (Fig. 6B, upper panel). Ki67 expression was dominantly suppressed in IR, AMD3100, whereas a combination of IR and AMD3100 samples. Meanwhile, cParp expression was significantly expressed through a combination of IR and AMD3100 sample, indicating the possible therapeutic enhancement of xenograft tumor (Fig. 6B, lower panel). Additionally, IHC of tissue microarray revealed that expression levels of CXCR4, p-STAT3, and Slug are much higher in NSCLC tissues than in normal tissues (Fig. 6C, upper and middle panels). Importantly, the expression of CXCR4 had a positive correlation with the expression of p-STAT3 and Slug within NSCLC tissues, thus indicating the possibility that CXCR4/STAT3/Slug pathway might be crucial for therapeutic resistance, including IR in NSCLC (Fig. 6C, lower panel).

Finally, to investigate the specific accumulation of ADM3100 on the CXCR4+ tumor cells, we radiolabeled it with ^64^Cu. The specific radioactivity of the [^64^Cu]AMD3100 after purification was typically 117 GBq/μmol (3.17 Ci/μmol), with radiochemical purity of >98% as determined by radio-ITLC. In A549 and A549-GR cells, the uptake of [^64^Cu]AMD3100 was 2.01 ± 0.42 %AD and 0.82 ± 0.20 %AD, respectively. Furthermore, receptor quantification analysis revealed that A549 and A549/GR expressed 1549.8 ± 378.0 and 3817.8 ± 793.8 receptors per cell, respectively, accounting for differences in [^64^Cu]AMD3100 binding. The 90-min PET/CT images in the lung tumor model showed selective accumulation of activity in A549/GR tumor compared with A549 tumor model. The transaxial images at 90 min showed significant uptake in the liver, kidney, bladder, and bone marrow (Fig. 6B). The data indicate that cell surface CXCR4 can be considered as a theragnostic target in CXCR4 high NSCLC cells.

## Discussion

Evidence indicates that despite availability of drastic interventions such as chemotherapy and radiotherapy certain cancer cells have been proven to cause formation of tumor bed and lead to poor prognosis. These cells have been established to have stem-like features. In the previous study, we have shown that CXCR4 is a functional CSC marker in NSCLC cells^13^. Since therapeutic resistance is whole mark of CSCs, we initially investigated the role of CXCR4 in the resistance to chemotherapeutic drugs and IR and found that there was no difference on drug resistance between CXCR4+ and CXCR4− cells, however we observed that A549/GR cells have much higher potential of resistance to IR^13^. In this study, we brought forward the role of CXCR4 in the IR resistance of NSCLC cells. Gefitinib resistant A549/GR cells showed higher clonogenic survival activity and led to more rapid resolution of γ-H2AX foci formation upon IR treatment rather than A549 parent cells. Via the gain and loss of function study, we ascertain that CXCR4 signaling is crucial for the resistance of NSCLC cells towards IR. In addition, we also found that STAT3 is critically involved in the CXCR4-mediated resistance of IR in NSCLC cells. Finally, microarray analysis of NSCLC cells revealed that Slug is a downstream effector molecule of CXCR4/STAT3 signaling in the IR resistance of NSCLC cells. Therefore, we conclude that CXCR4/STAT3/Slug signaling axis has functional role in the IR resistance as well as maintenance of stemness in NSCLC cells.

Many studies have shown that CXCR4 is one of the markers for cancer stem cells in various types of cancer^12,19,20^. Our and others’ research also discovered that high level of CXCR4 expression is also found in stem-like lung cancer cells^13,20^. Since CSCs are well known as the causative cells of therapeutic resistance, we examined whether the crucialness of CXCR4 signaling for drug and IR resistance towards NSCLC cells. Using drug resistant A549/GR cells, we found the CXCR4 signaling is deeply involved in the resistance to IR, but not in drug resistance. Through knockdown and overexpression studies of CXCR4 in NSCLC cells, we deduced the important role of CXCR4 signaling in IR resistance of NSCLC cells. In line with our results, some reports have revealed that CXCR4 is a CSC marker and at the same time, an IR resistant marker^21–23^. Therefore, as far as we know, this is the first evidence suggesting the CXCR4 can be a good target for enhancing radiosensitivity of NSCLC.

In the previous study, we found that CXCR4 preferentially signaled to STAT3 for the maintenance of stemness in NSCLC cells^13^. Several reports have shown that STAT3 signaling is involved in radiotherapeutic and chemotherapeutic sensitivity in NSCLC^24–26^. Therefore, in this study, we investigated whether STAT3 affects IR resistance of NSCLC cells or not. siRNA-mediated knockdown and pharmacologic inhibition of STAT3 indicated that CXCR4-mediated activation of STAT3 is crucial for IR resistance in NSCLC cells. Interestingly, phosphorylation of STAT3 at the tyrosine 705 (pY-STAT3) but not at the serine 727 sites (pS-STAT3) seemed to play an important role for IR resistance in NSCLC cells. Overall, our results and others suggest that STAT3 is a good target to increase radiotherapy effect of NSCLC.

In the present work, we found that the transcription factor Slug is deeply involved in the IR resistance of NSCLC cells as a downstream effector molecule for CXCR4/STAT3 signaling. The fact that Slug is involved in IR resistance has been studied in various types of cancers^27–32^. As a transcriptional repressor, Slug protects cells by inhibiting the expression of puma, a Bcl-2 family proapoptotic protein, from p53-mediated apoptosis caused by DNA damage such as IR exposure^30,33^. In addition, recent report have shown that deficient of Slug leads to accumulation of DNA damage by impairing the recruitment of RAD51 at the damaged site^34^. Therefore, it is highly probable that CXCR4/STAT3 signaling augments the expression of Slug which suppresses puma expression and enhances the resolution of DNA damage by IR in NSCLC cells. Although the precise molecular intricacies of these effects in tumor could be more complex and involve cooperative functioning of other molecules and could partly be related to IR resistance, our findings definitely suggest that Slug is a critical determinant of CXCR4-mediated IR resistance in these cells. Although we have focused on these phenomena in lung cancer, our findings can possibly be extended to other cancers to represent a common, yet underestimated, mechanism that could make cancer cells difficult to eradicate.

Summing up, this study observes that CXCR4/STAT3/slug signaling is critical in maintaining IR resistance of NSCLC cells, and offers the possibility of targeting CXCR4 signaling in suppressing and eliminating CSCs of NSCLC by IR. Therefore, blockade of CXCR4/STAT3 pathway could be a promising approach for the efficient sensitizing NSCLC cells to IR, hence being useful for the radiotherapy of this devastating cancer.

## Supplementary information

supple fig1

supple fig2

supple fig3

supple fig4

supple fig5

supple fig6

supple fig7

supple legend
