# Supplementary figures and images for: CXCR4 uses STAT3-mediated slug expression to maintain radioresistance of non-small cell lung cancer cells: emerges as a potential prognostic biomarker for lung cancer
Source: Cell Death Dis. 2021 Jan 7;12(1):48. doi: 10.1038/s41419-020-03280-5 (PMC7791104; doi:10.1038/s41419-020-03280-5)

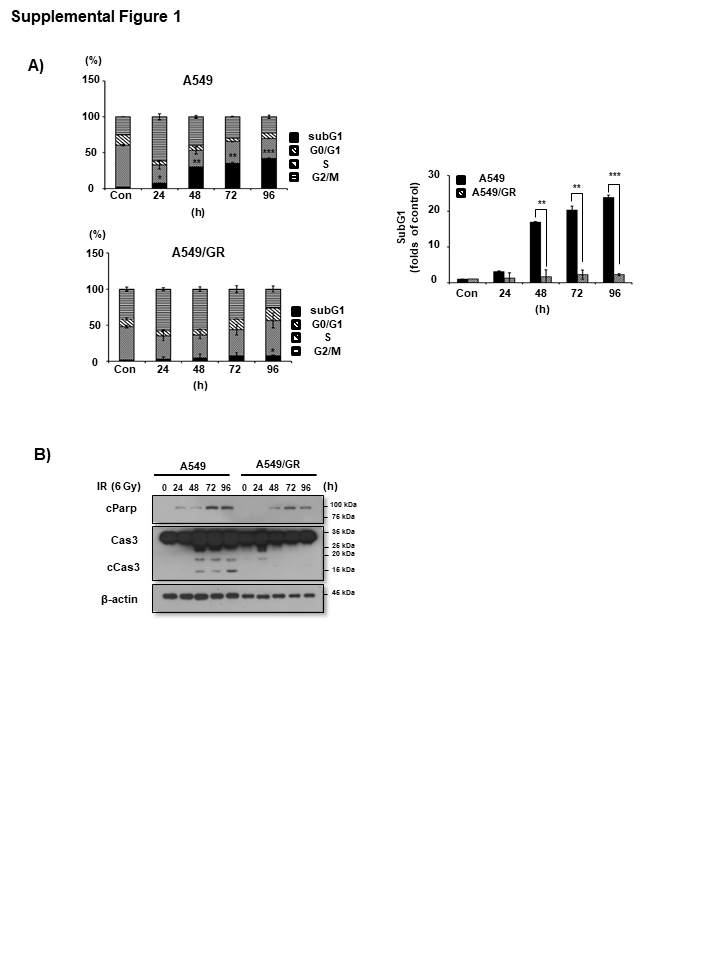

Supplement: Supplementary file 1 — supple fig1 [file 41419_2020_3280_MOESM1_ESM.tif]

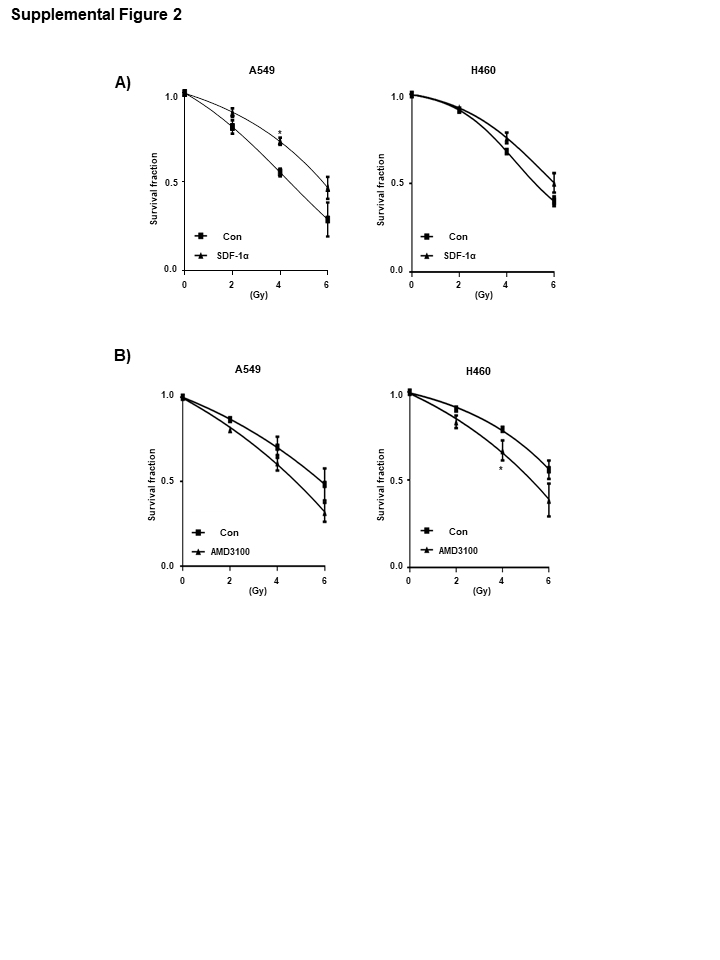

Supplement: Supplementary file 2 — supple fig2 [file 41419_2020_3280_MOESM2_ESM.tif]

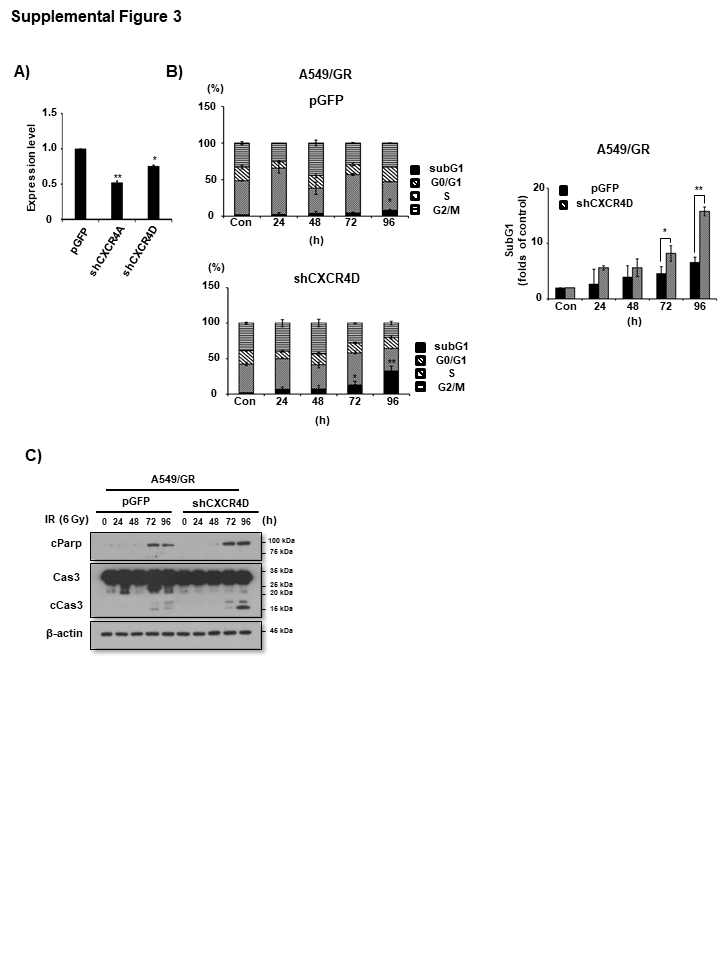

Supplement: Supplementary file 3 — supple fig3 [file 41419_2020_3280_MOESM3_ESM.tif]

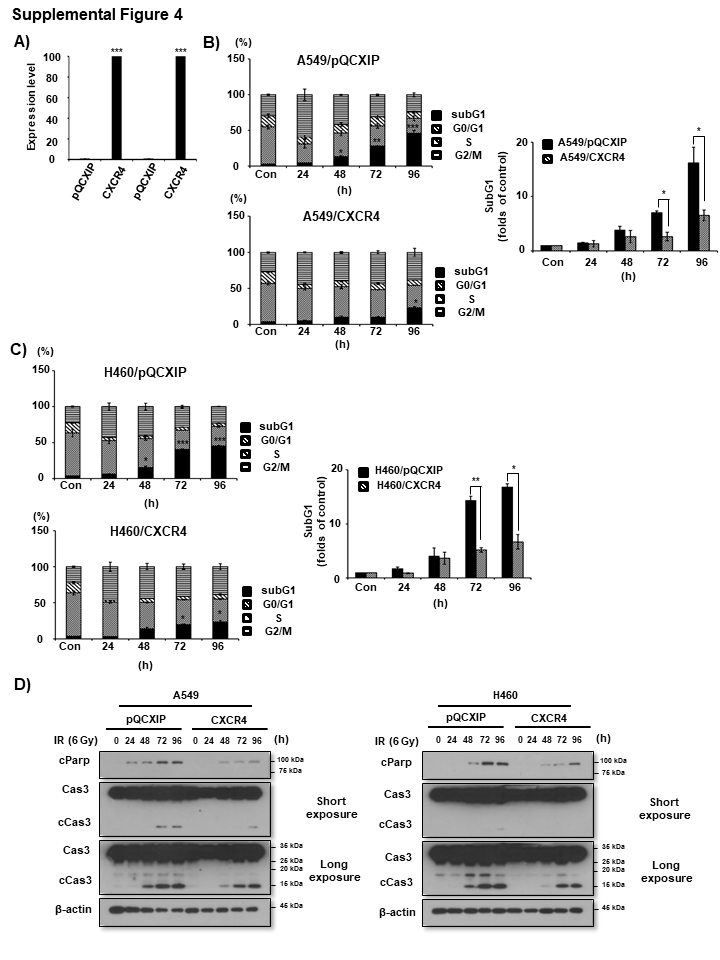

Supplement: Supplementary file 4 — supple fig4 [file 41419_2020_3280_MOESM4_ESM.tif]

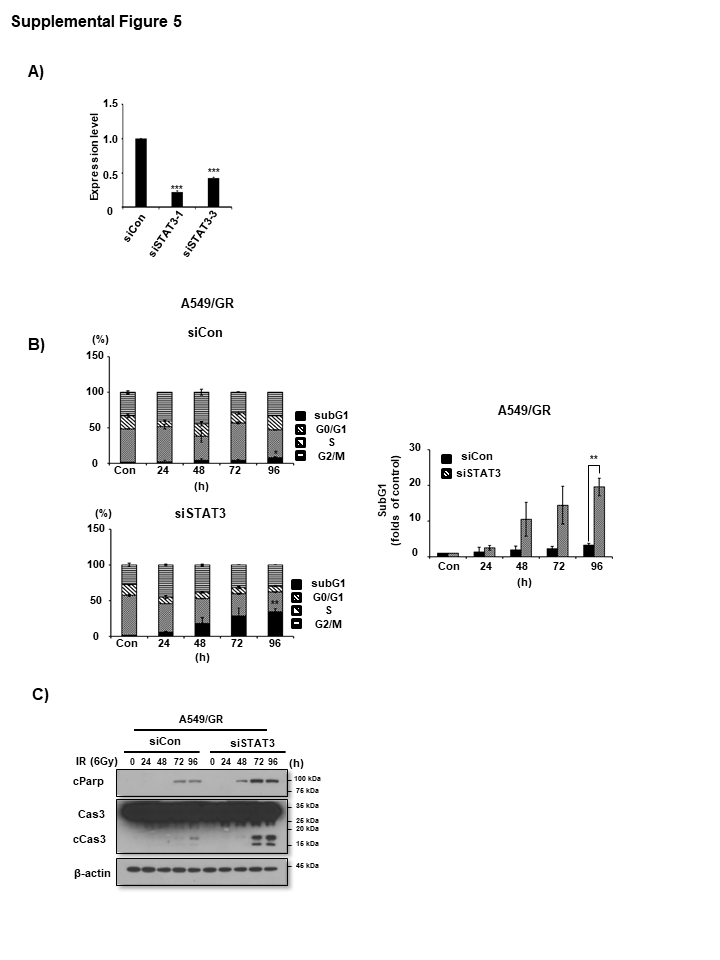

Supplement: Supplementary file 5 — supple fig5 [file 41419_2020_3280_MOESM5_ESM.tif]

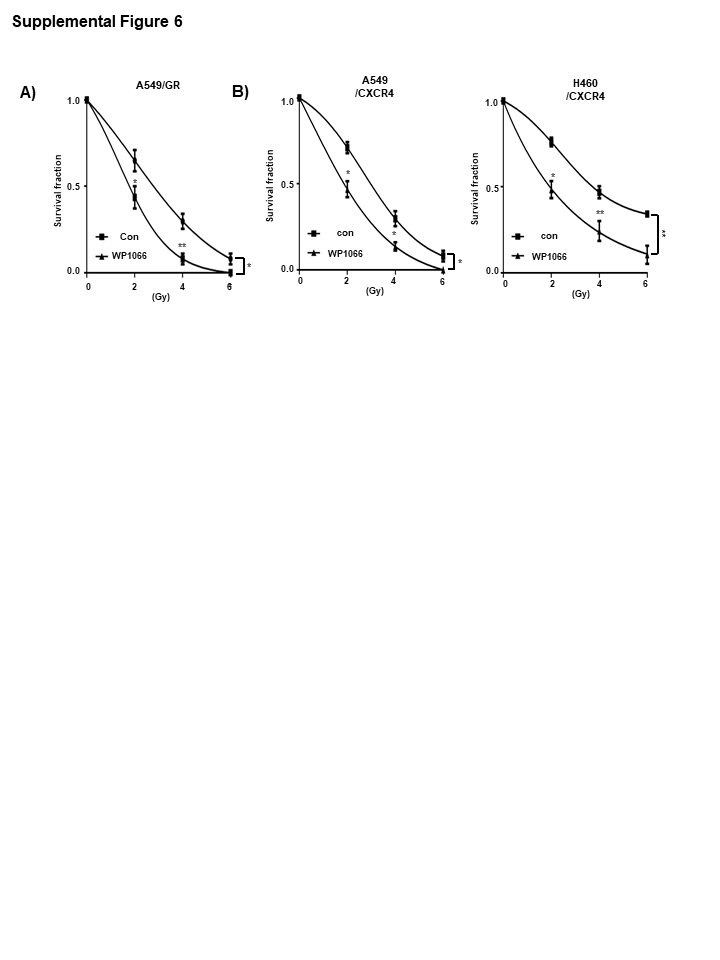

Supplement: Supplementary file 6 — supple fig6 [file 41419_2020_3280_MOESM6_ESM.tif]

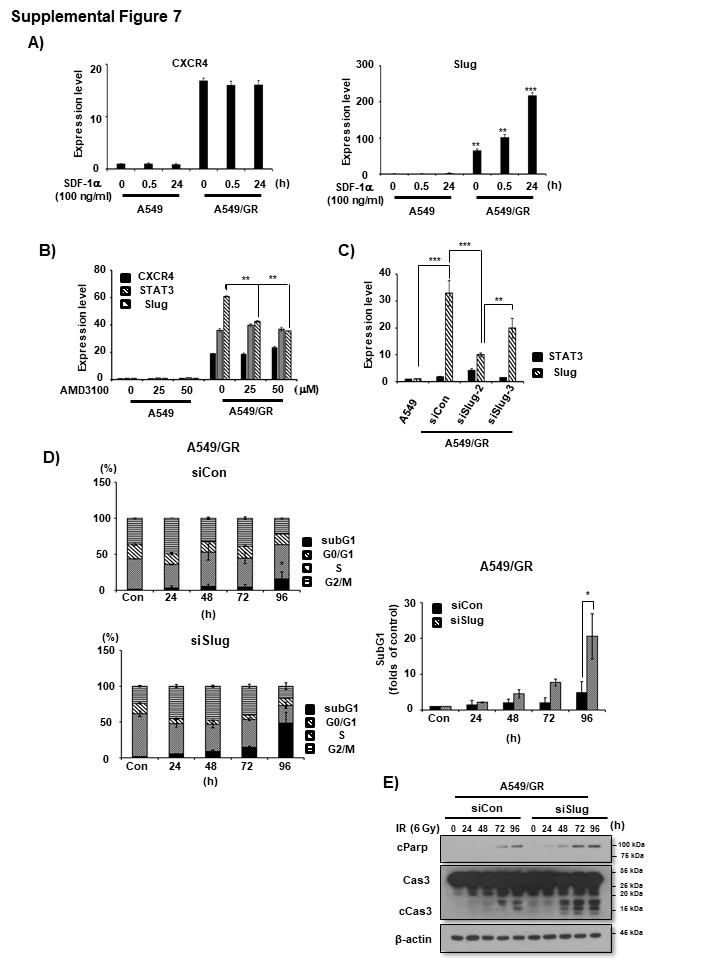

Supplement: Supplementary file 7 — supple fig7 [file 41419_2020_3280_MOESM7_ESM.tif]
