# Supplementary material for: CXCR4 uses STAT3-mediated slug expression to maintain radioresistance of non-small cell lung cancer cells: emerges as a potential prognostic biomarker for lung cancer
Source: Cell Death Dis. 2021 Jan 7;12(1):48. doi: 10.1038/s41419-020-03280-5 (PMC7791104; doi:10.1038/s41419-020-03280-5)
Supplement: Supplementary file 8 — supple legend [file 41419_2020_3280_MOESM8_ESM.docx]

Supplemental Figure legends

**Supplemental Figure 1**. IR induces more cell death in A549 cells than A549/GR cells. (A) Cell cycle and (B) Western blot analysis of A549 and A549/GR cells after exposed to IR (6 Gy) at various time points indicated in the Figure. Histogram of the cell cycle analysis (A, left) and quantification of the subG1 fractions (A. right). cParp, cleaved Parp; Cas3, caspase 3; cCas3, cleaved caspase 3. **p*<0.05, ***p*<0.01, ****p*<0.005.

**Supplemental Figure 2**. CXCR4 signaling also affects clonogenic survival of NSCLC cells after IR. IR clonogenic survival assay of NSCLC cells (A549 and H460) pretreated with or without SDF-1α (100 ng/ml) (A) and AMD3100 (10 μM) (B), followed by exposed to IR in a dose-dependent manner indicated in Figure. Cells (2.5 × 10^2^) were seeded in 60 mm dishes. Next day, cells were exposed to IR in a dose indicated in the Figure. **p*<0.05.

**Supplemental Figure 3**. Knockdown of CXCR4 increases cell death in A549/GR cells to IR. (A) qRT-PCR analysis of CXCR4 in A549/GR cells transfected with shControl (pGFP) or shCXCR4A and D. (B) Cell cycle and (C) Western blot analysis of shControl (pGFP)- or shCXCR4D-transfected A549/GR cells after exposed to IR (6 Gy) at various time points indicated in the Figure. Histogram of the cell cycle analysis (B, left) and quantification of the subG1 fractions (B. right). cParp, cleaved Parp; Cas3, caspase 3; cCas3, cleaved caspase 3. **p*<0.05, ***p*<0.01.

**Supplemental Figure 4**. Overexpression of CXCR4 decreases cell death in NSCLC to IR. (A) qRT-PCR analysis of NSCLC cells (A549 and H460) transfected with CXCR4. (B, C) Cell cycle and (D) Western blot analysis of control vector (pQCXIP) and CXCR4 overexpressing (CXCR4) NSCLC cells (A549 and H460) after exposed to IR (6 Gy) at various time points indicated in the Figure. Histogram of the cell cycle analysis (B and C, left) and quantification of the subG1 fractions (B and C. right). cParp, cleaved Parp; Cas3, caspase 3; cCas3, cleaved caspase 3. **p*<0.05, ***p*<0.01, ****p*<0.005.

**Supplemental Figure 5**. Knockdown of STAT3 increases cell death in A549/GR cells to IR. (A) qRT-PCR , (B) cell cycle, and (C) Western blot analysis of siControl (siCon)- or siSTAT3-transfected A549/GR cells after exposed to IR (6 Gy) at various time points indicated in the Figure. Histogram of the cell cycle analysis (B and C, left) and quantification of the subG1 fractions (B and C. right).

**Supplemental Figure 6.** STAT3 inhibitor suppresses clonogenic survival of NSCLC cells after IR. IR clonogenic survival assay of NSCLC cells (A549/GR (A), A549/CXCR4 (B) and H460/CXCR4 (C)) pretreated with or without WP1066 (5 μM), followed by exposed to IR in a dose-dependent manner indicated in Figure. Cells (2.5 × 10^2^) were seeded in 60 mm dishes. Next day, cells were exposed to IR in a dose indicated in the Figure. **p*<0.05, ***p*<0.01.

**Supplemental Figure 7**. Knockdown of Slug increases cell death in A549/GR cells to IR. (A-C) qRT-PCR analysis of A549 or A549/GR cells treated with SDF-1a (A), AMD3100 (B), and transfected with of sicontrol (siCon) or siSlug (C). (D) Cell cycle and (E) Western blot analysis of A549/GR cells transfected with siRNA described in the Figure. Cells were exposed to IR (6 Gy) at various time points indicated in the Figure. Histogram of the cell cycle analysis (D, left) and quantification of the subG1 fractions (D. right). **p*<0.05, ***p*<0.01, ****p*<0.005.
